# Incentive Spirometer in COVID-19: A Systematic Review

**DOI:** 10.3390/jcm15041425

**Published:** 2026-02-11

**Authors:** Marina Kloni, Alexandros Heraclides, Theognosia Panteli, Alexios Klonis, Panagiotis Rentzias, Christos Karagiannis

**Affiliations:** 1 Physiotherapy Department, Medical Center EDEM, Tersefanou, 7562 Larnaca, Cyprus; 2 Department of Health Sciences, European University of Cyprus, 6 Diogenous Str, Engomi, 2404 Nicosia, Cyprus; 3 Department of Anaesthesiology, The Newcastle Upon Tyne Hospitals NHS Foundation Trust, Newcastle NE7 7DN, UK

**Keywords:** incentive spirometer, COVID-19, Post-COVID-19 syndrome, systematic review, respiratory physiotherapy, rehabilitation

## Abstract

**Background/Objectives:** COVID-19 and its sequelae have affected millions worldwide, with many individuals experiencing persistent symptoms such as dyspnea, fatigue and reduced quality of life. Respiratory physiotherapy is commonly used to support patients with pulmonary conditions. This systematic review aimed to evaluate the effects of the incentive spirometer on cardiopulmonary, functional and patient-reported outcomes in adults during the acute and post-COVID-19 phases. **Methods:** A systematic literature search was conducted in PubMed, CINAHL, Scopus, Clinical Trials.gov and Google Scholar to identify studies published between January 2020 and April 2025. Owing to substantial heterogeneity in study design, populations, interventions and outcome measures, quantitative synthesis was not feasible and findings were synthesized narratively. **Results**: Twelve studies involving 573 participants were included. Within-group analyses showed improvements in pulmonary outcomes (including FEV_1_, FVC, and oxygen saturation), reductions in dyspnea, and improvements in quality of life following incentive spirometer. Improvements in pulmonary function were reported primarily in post-COVID-19 populations, whereas reductions in anxiety and improvements in quality of life were reported mainly in acute COVID-19 settings. Between-group comparisons demonstrated statistically significant differences in favor of the incentive spirometer for selected pulmonary and functional outcomes (including FVC, DLCO, oxygen saturation, six-minute walk test, and 30 s sit-to-stand test), while no significant differences were observed for other outcomes such as peak expiratory flow, respiratory rate, or heart rate variability. Randomized controlled trials were judged to have a moderate risk of bias, non-randomized studies a moderate-to-serious risk, and certainty of evidence ranged from very low to moderate. **Conclusions:** Incentive spirometer may support respiratory, functional, and psychological recovery in adults during the acute and post-COVID-19 phases. However, effects vary across outcomes and comparator interventions, and the overall certainty of evidence is low to moderate. Further high-quality research is required to confirm effectiveness and guide optimal clinical use.

## 1. Introduction

Coronavirus disease 2019 (COVID-19) is a highly contagious disease caused by Severe Acute Respiratory Syndrome Coronavirus 2 (SARS-CoV-2). First identified in Wuhan, China, in December 2019, it rapidly spread worldwide, leading the World Health Organization to declare a Public Health Emergency of International Concern (PHEIC) in January 2020 and a pandemic in March 2020 [1]. Clinical manifestations of COVID-19 are highly heterogeneous, ranging from asymptomatic infection to severe respiratory failure, Acute Respiratory Distress Syndrome (ARDS), and death [2,3]. SARS-CoV-2 infection induces pulmonary inflammation and dysregulated immune responses, resulting in diffuse alveolar damage and, in some cases, hypoxic respiratory failure [2]. Radiological abnormalities such as ground-glass opacities and fibrotic changes may persist after infection and are associated with impaired pulmonary function, reduced exercise capacity and prolonged dyspnea [4,5,6,7,8,9,10,11]. Common signs and symptoms include dry cough, dyspnea, fatigue, fever, anosmia, ageusia and myalgia [2,12,13,14].

Post-COVID-19 syndrome (PCS) is a recognized complication of COVID-19 infection, although its true prevalence remains difficult to estimate due to heterogeneity in study designs and follow-up durations. Reported prevalence ranges from 6.2% to 53%, depending on disease severity, hospitalization status, intensive care admission, and time since infection [15,16,17]. According to current definitions, PCS is diagnosed when symptoms persist for at least two months and are present three months after the acute infection, without an alternative diagnosis [18,19]. PCS affects multiple organ systems and is commonly characterized by fatigue, dyspnea, brain fog, cognitive impairment, joint pain, anxiety, depression, sleep disturbances and chest pain, leading to substantial reductions in quality of life [7,9,11,20,21,22,23,24,25,26].

Beyond its clinical consequences, COVID-19 has placed a substantial burden on healthcare systems and economies worldwide. While millions of individuals have died from the disease [27], an even larger population continues to experience long-term physical, psychological, social and economic consequences following recovery from the acute infection [7,9,11,22,23,24,25,26]. In the United States, Post-COVID-19 healthcare utilization has increased significantly, with additional monthly medical expenditures estimated at $223.59 per patient [28]. Broader societal impacts have also been reported, including substantial losses in quality-adjusted years and associated economic costs [29]. Although these estimates are country-specific, they highlight the potential global burden of PCS and underscore the importance of effective rehabilitation strategies.

Respiratory physiotherapy has been recommended during both the acute and recovery phases of COVID-19 to address respiratory symptoms and functional impairments [30,31,32]. Evidence suggests that respiratory physiotherapy may improve dyspnea, fatigue and physical function in individuals recovering from COVID-19 [33,34,35]. Among the techniques used, the incentive spirometer is a simple handheld device designed to promote slow, deep and sustained inspirations through visual feedback, mimicking physiological signs or yawns [36,37,38]. These breathing patterns are intended to support lung expansion and ventilation [36,37,38].

Incentive spirometer is widely used in clinical practice, particularly in postoperative care and chronic respiratory conditions, to improve lung function [37,39,40,41,42,43]. However, its use in patients with COVID-19 has been controversial. Some pandemic guidelines did not recommend routine use, citing concerns related to aerosol generation, increased work of breathing, potential exacerbation of respiratory distress and limited support evidence [30,44]. In contrast, other guidelines supported its selective use when clinically indicated, emphasizing its potential role as a contactless intervention that should be discontinued if adverse effects such as coughing occurred [45,46]. Additional considerations include the widespread use and economic impact of the incentive spirometer, with estimated annual expenditures in the United States exceeding $949.4 million [47].

Given the persistent prevalence of Post-COVID-19 respiratory dysfunction, the widespread clinical use of the incentive spirometer and its associated economic implications, a comprehensive evaluation of its effectiveness is warranted. The primary aim of this systematic review was to assess the effects of the incentive spirometer on pulmonary function outcomes in adults during the acute and Post-COVID-19 phases. Secondary aims were to evaluate its effects on respiratory symptoms, functional capacity, psychological outcomes, quality of life and to compare its effectiveness with standard care and deep breathing interventions.

## 2. Materials and Methods

### 2.1. Protocol Registration and Guidelines

This systematic review was conducted in accordance with the Preferred Reporting Items for Systematic Reviews and Meta-Analyses (PRISMA) guidelines [48] and the Synthesis Without Meta-analysis (SWiM) reporting framework [49]. The review protocol was prospectively registered with the International Prospective Register of Systematic Reviews (PROSPERO; registration number: CRD42023468835; 19 August 2024).

The registered objectives focused on adults with Post-COVID-19 syndrome, evaluating whether incentive spirometry improves pulmonary function compared with other interventions or no intervention. For the present review, the scope was clarified prior to study selection and data extraction to include adults in both the acute and Post-COVID-19 phases, reflecting the overlapping clinical use of incentive spirometry. No changes were made to the predefined outcomes, study selection procedures, or data extraction methods.

### 2.2. Sources and Search Strategy

A systematic literature search was conducted in PubMed, PEDro, CINAHL, and Scopus for studies published between January 2020 and April 2025. Two reviewers (M.K. and T.P.) independently performed the searches. Additional grey literature searches were conducted using Google Scholar (https://scholar.google.com/ accessed on 6 February 2026) and ClinicalTrials.gov (https://clinicaltrials.gov/ accessed on 6 February 2026). Reference lists of all included studies were manually screened to identify further eligible publications. The final search was conducted on 30 April 2025. Detailed search strategies, including keywords and Boolean operators for each database, are provided in Table 1.

### 2.3. Study Selection

**Figure 1:**
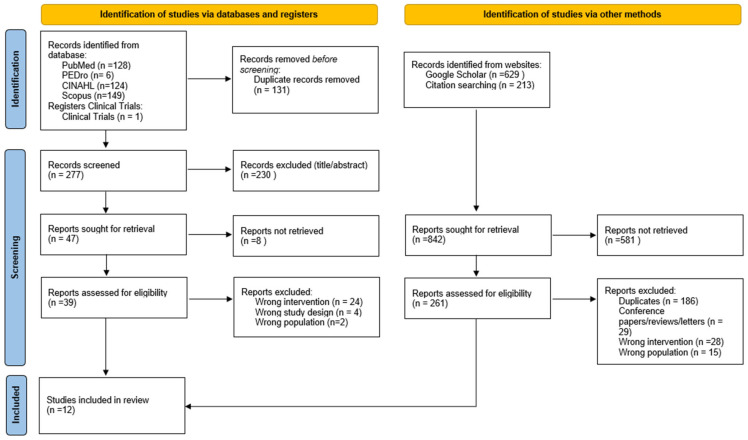
PRISMA flow diagram of the study selection process from database searches and other sources.

After removal of duplicate records, titles and abstracts were independently screened by two reviewers (M.K. and T.P). Full-text articles deemed potentially eligible were assessed against the predefined inclusion and exclusion criteria. Discrepancies were resolved through discussion or consultation with a third reviewer (C.K.). All reviewers were proficient in English reading and comprehension, ensuring accurate extraction and assessment of study data. The study selection process is summarized in a PRISMA flow diagram (Figure 1).

### 2.4. Eligibility Criteria and Research Question

Eligibility was defined using the PICOS (Participants, Intervention, Comparison, Outcomes and Study design) framework:

1. Participants (P): Adults (≥18 years) with a confirmed COVID-19 infection, including both acute and Post-COVID-19 phases.
2. Intervention (I): Incentive spirometer, used alone or with other physiotherapy interventions.
3. Comparison (C): Standard care or alternative physiotherapy interventions.
4. Outcomes (O): Pulmonary function, respiratory symptoms (such as dyspnea), functional capacity, psychological outcomes and quality of life.
5. Study Design (S): Interventional studies, including randomized controlled trials, clinical trials, cohort studies, case studies and observational studies.
6. Language: English or Greek.

Exclusion criteria included reviews, editorials, letters, commentaries, conference abstracts and studies without full-text availability.

Research Question: In adults with COVID-19 or Post-COVID-19 syndrome, does incentive spirometer compared with usual care or other physiotherapy interventions, improved pulmonary function and other health-related outcomes?

### 2.5. Data Extraction

Two reviewers (M.K. and T.P.) independently extracted data using a standardized collection form. Extracted information included: first author, year, country, sample size, participant age, COVID-19 phase (acute or Post-COVID-19), study design, intervention and comparator details, outcomes assessed, intervention duration and frequency, and main findings. Disagreements were resolved through discussion or consultation with a third reviewer (C.K.).

### 2.6. Risk of Bias Assessment

Risk of bias was independently assessed by two reviewers (M.K. and T.P.) in accordance with the Cochrane Handbook for Systematic Reviews of Interventions, using the recommended risk of bias assessment tools [50,51,52]. Disagreements were resolved through discussion or consultation with a third reviewer (C.K.).

The randomized controlled trials (RCTs) were assessed using the Revised Cochrane Risk of Bias tool (RoB 2, version 22 August 2019), evaluating five domains: randomization process, deviations from intended interventions, missing outcome data, measurement of outcomes and selection of reported results [50]. Each domain was rated as low risk, some concerns, or high risk, leading to an overall judgment.

The non-randomized studies were assessed using the Risk Of Bias In Non-randomized Studies of Interventions (ROBINS-I, Version 2) tool across seven domains: confounding, classification of interventions, selection of participants into the study/analysis, deviations from intended interventions, missing data, outcome measurement and selection of reported results [51]. Each domain was rated as low, moderate, serious or critical risk, informing an overall risk of bias judgment.

## 3. Results

### 3.1. Study Selection and Characteristics

A total of 1250 potentially relevant articles were identified through database searches and manual reference screening. After screening, 12 studies met the eligibility criteria and were included (Figure 1). The 12 studies involved 573 participants, with sample sizes ranging from 11 to 160 and a mean age between 22.7 and 65.8 years. Four studies included participants in the acute phase of COVID-19 [53,54,55,56], and eight studies included Post-COVID-19 individuals [57,58,59,60,61,62,63,64]. Studies were conducted across five countries, most commonly in Asia (*n* = 4) and Africa (*n* = 1). Detailed study characteristics are presented in Table 2.

### 3.2. Intervention and Comparators

Intervention approaches varied substantially across studies. Six studies investigated a flow-oriented spirometer [53,56,57,58,59,60]. Four studies used the incentive spirometer in a seated position [54,58,62,63]. Eight studies implemented interventions over four weeks [56,57,58,59,61,62,63,64]. Five studies were conducted in a hospital setting [53,54,55,56,57], and the remaining in outpatient or community-based environments (Table 3). Comparator interventions consisted of diaphragmatic breathing exercises [55,58,61,62,63,64] or standard care [53,54,56,57,59,60].

### 3.3. Effects of Incentive Spirometer

Due to substantial heterogeneity across the included studies in terms of outcome measures, assessment tools, study designs, comparator interventions and intervention protocols, quantitative synthesis through meta-analysis was not feasible. Consequently, a narrative synthesis was performed. The results were structured according to the predefined outcomes and are presented across four main domains: (1) primary outcomes including cardiopulmonary function and dyspnea, (2) secondary outcomes including functional performance, quality of life, anxiety and depression (3) COVID-19 disease phase and (4) incentive spirometer versus comparator interventions. All outcome data, including baseline and post-intervention values, comparator group results and between-group comparisons, are summarized in Table 4.

#### 3.3.1. Primary Outcomes: Cardiopulmonary Function and Dyspnea

Within-group changes following incentive spirometer use demonstrated improvements across several cardiopulmonary outcomes (Table 4). Pulmonary function measures, including forced vital capacity (FVC), forced expiratory volume in one second (FEV_1_), FEV_1_/FVC ratio, diffusing capacity for carbon monoxide (DLCO), slow vital capacity (SVC), maximal voluntary ventilation (MVV), peak expiratory flow (PEF), and peak cough flow (PCF), showed improvements from baseline to post-intervention in studies reporting these outcomes [57,58,60,62]. Improvements in oxygen saturation and reductions in respiratory rate and dyspnea scores were also reported in participants with mild to moderate symptoms [53,60]. Additionally, one study reported increases in diaphragmatic thickness and excursion following the intervention [60]. In contrast, heart rate variability parameters, including RMSSD, SDNN, and LF/HF ratio, showed no meaningful within-group changes [61].

#### 3.3.2. Secondary Outcomes: Functional Performance, Quality of Life, Anxiety and Depression

Functional performance improved following incentive spirometer use, with increases reported in the six-minute walk test (6MWT), six-minute step test (6MST), four-meter gait time test (4MGT), and 30 s sit-to-stand test (30STS) (Table 4) [55,60,63,64]. Improvements in quality of life were observed in one study conducted during the acute phase of COVID-19 [53]. Reductions in anxiety scores were reported in two studies [53,59], while one study also demonstrated a reduction in depressive symptoms following the intervention. No differences were observed for mortality, intubation, or intensive care unit admission rates [54].

#### 3.3.3. Outcomes According to COVID-19 Disease Phase

Among participants in the acute phase of COVID-19, incentive spirometer use was associated with improvements in oxygen saturation, respiratory rate, dyspnea, anxiety, quality of life, and functional performance outcomes [53,54,55,56]. In the Post-COVID-19 phase, improvements were more consistently reported for pulmonary function measures, oxygen saturation, dyspnea, psychological outcomes, length of hospital stay, and functional capacity, including walking and step-based tests [57,58,59,60,61,62,63,64] (Table 4).

#### 3.3.4. Incentive Spirometer Compared with Comparator Interventions

This section presents between-group comparisons of incentive spirometer use versus comparator interventions, highlighting differences in pulmonary, functional, and patient-reported outcomes as reported in the included studies (Table 4).

When compared with diaphragmatic breathing exercises, incentive spirometer use demonstrated mixed effects. Several studies reported no between-group differences in pulmonary or autonomic outcomes, while others favored incentive spirometer use for functional performance and cough-related measures. Comparisons with standard care also yielded variable results. Some studies reported greater improvements with incentive spirometer use in pulmonary function, oxygenation, dyspnea, quality of life, diaphragm function, and walking capacity, whereas other outcomes did not differ between groups (Table 4).

#### 3.3.5. Risk of Bias of Included Studies

Risk of bias was assessed for all included studies. Among the seven RCTs, all reported random allocation of participants; however, methodological details were frequently incomplete [58,63], and allocation concealment was often not reported [53,57,58,63], raising concerns regarding selection bias. Blinding of participants and therapists was generally not feasible due to the nature of the intervention, whereas blinding of outcome assessors was reported in several studies [54,57,59,60]. Incomplete reporting of dropouts or losses to follow-up introduced potential attrition bias in some trials [53,59]. Overall, the RCTs were judged to have a moderate risk of bias, primarily due to concerns related to the randomization process, deviations from intended interventions, and outcome measurement. The four pre–post studies and one observational study were judged to have moderate to serious risk of bias, mainly due to potential confounding [61,62,64], selection bias, lack of blinding, measurement bias, and missing data [56,61,62,64]. Detailed risk-of-bias assessments for the studies are provided in Figures S1 and S2 in the Supplementary Materials.

#### 3.3.6. Certainty of Evidence

The certainty of evidence was assessed using the GRADE approach. Overall, the certainty ranged from very low to moderate across outcomes. Pulmonary function outcomes were rated as low to moderate certainty due to risk of bias in non-randomized studies, heterogeneity, and modest sample sizes. Dyspnea and functional performance outcomes were judged to have moderate certainty. Evidence for anxiety, depression, and quality-of-life outcomes was judged to have low to very low certainty, primarily due to limited sample sizes and variability in outcome reporting. Evidence for heart rate variability outcomes was of very low certainty, as it was derived from a single small pre–post study. A GRADE summary is provided in Table S1 in the Supplementary Materials.

## 4. Discussion

This systematic review evaluated the effects of the use of the incentive spirometer in adults during the acute and Post-COVID-19 phases, with a focus on pulmonary function, dyspnea, functional performance, psychological outcomes and quality of life. The review addressed a clinically relevant question given the persistent respiratory and functional impairments reported after COVID-19 infection. Overall, the findings suggest that incentive spirometer use may be associated with improvements across several respiratory and patient-centered outcomes; however, the magnitude, consistency and certainty of these effects varied across outcomes, study designs and comparator interventions.

### 4.1. Cardiopulmonary Outcomes and Dyspnea

Improvements in pulmonary function and dyspnea were among the most frequently reported findings in studies evaluating incentive spirometer use. These effects may be explained by the deep, sustained inspirations encouraged by the device, which promote lung expansion, alveolar recruitment, and improved ventilation distribution [65]. Such breathing patterns may also enhance diaphragmatic engagement and reduce rapid, shallow breathing, potentially contributing to reductions in respiratory rate and perceived dyspnea [66,67,68,69]. These mechanisms are consistent with evidence from other respiratory conditions, including acute respiratory distress syndrome, pulmonary fibrosis, and thoracic trauma [67,69,70,71].

Despite improvements in respiratory parameters, no significant changes were observed in cardiac autonomic outcomes, such as heart rate variability, in the limited evidence available. The absence of detectable effects on these parameters may be influenced by psychological stress, reduced functional capacity, short intervention duration, or insufficient statistical power [61]. Given that heart rate variability was assessed in only one small pre–post study, no firm conclusions can be drawn regarding autonomic effects.

### 4.2. Functional Performance, Quality of Life, Anxiety and Depression

Psychological outcomes, including anxiety and depression, were assessed in a limited number of randomized controlled trials. Incentive spirometer use may contribute to psychological benefits through controlled breathing, visual biofeedback, and increased perceived control over breathing and recovery [72,73,74,75]. Improvements in respiratory symptoms may also indirectly support psychological well-being by enhancing sleep, mobility, and daily functioning. However, evidence for psychological outcomes remains limited and is based primarily on within-group changes, warranting cautious interpretation.

Quality-of-life improvements were reported in a small number of studies, primarily during the acute phase of COVID-19. These changes may reflect combined improvements in respiratory symptoms, functional capacity, and psychological status [76,77,78]. Nonetheless, the low certainty of evidence and the limited number of trials prevent definitive conclusions regarding the impact of incentive spirometer use on quality of life.

Several studies reported improvements in functional performance following incentive spirometer use, including gains in walking distance, step tests, gait speed, and sit-to-stand performance. Improved respiratory mechanics and ventilatory efficiency may reduce exertional dyspnea and lower the ventilatory demand during physical activity, thereby facilitating better performance on functional tests [79,80]. These functional improvements are clinically relevant, particularly for individuals recovering from COVID-19 who frequently report exercise intolerance and reduced mobility.

### 4.3. Other Outcomes

Incentive spirometer use did not demonstrate statistically significant effects on severe clinical outcomes such as mortality, intubation, or intensive care unit admission. These outcomes are influenced by multiple factors, including disease severity, comorbidities, and broader medical management, which may limit the impact of isolated respiratory interventions. In contrast, reductions in length of hospital stay observed in some studies may reflect improved respiratory function, reduced dyspnea, and faster recovery of functional capacity, potentially facilitating earlier discharge.

### 4.4. Incentive Spirometer as a Single or Multimodal Intervention

Comparisons between incentive spirometer use and other interventions, including diaphragmatic breathing and standard care, yielded mixed results. In some cases, incentive spirometer use resulted in greater improvements in pulmonary or functional outcomes, while in others, no significant differences were observed. These findings suggest that incentive spirometer use alone may provide limited benefits for certain outcomes and may be more effective when integrated into multimodal respiratory or rehabilitation programs. Multicomponent interventions are generally considered more comprehensive, as they address the multiple impairments commonly observed in COVID-19 and Post-COVID-19 syndrome, including muscle weakness, fatigue, balance deficits, and reduced endurance [81,82].

### 4.5. Sources of Heterogeneity and Inconsistent Findings

The variability in findings across studies may be attributed to several factors, including small sample sizes, short intervention durations, differences in COVID-19 severity, vaccination status, and patient adherence. Variations in intervention protocols—such as device type (flow- versus volume-oriented), frequency, duration, and supervision—may also influence outcomes [80,83,84,85,86,87]. Additionally, diaphragmatic breathing exercises often share similar physiological mechanisms to the incentive spirometer, potentially resulting in comparable effects and reducing detectable between-group differences. The heterogeneity observed across studies contributed to the inability to perform meta-analysis and limited the generalizability of the findings.

### 4.6. Feasibility and Compliance

Incentive spirometer use appeared feasible across inpatient, outpatient, and home-based settings [70,71,88,89]. However, adherence was inconsistently monitored, and poor compliance may have attenuated observed effects. Previous evidence [90] suggests that improper technique, insufficient frequency of use, and lack of supervision are common barriers. Structured education, follow-up, and adherence monitoring may enhance the effectiveness of incentive spirometer interventions, particularly in unsupervised settings.

### 4.7. Comparison with Previous Reviews

Previous reviews examining incentive spirometer use in COVID-19 populations have reported inconsistent conclusions. While one identified potential improvements in respiratory and psychological outcomes [91], the other concluded that evidence was insufficient due to heterogeneity and methodological limitations [92]. The present review expands upon earlier work by including a broader range of outcomes and study designs while highlighting the same underlying limitations, reinforcing the need for more robust evidence.

### 4.8. Risk of Bias in Included Studies

The interpretation of findings is influenced by the methodological quality of the included studies. Randomized controlled trials were generally judged to have a moderate risk of bias, primarily related to incomplete reporting of randomization procedures and lack of blinding. Non-randomized studies were subject to moderate-to-serious risk of bias due to confounding, selection bias, and missing data. These limitations were reflected in the GRADE assessments, which ranged from very low to moderate certainty across outcomes. Consequently, while some outcomes show promising trends, the overall strength of evidence remains limited.

### 4.9. Limitations and Future Research

Several limitations in this review restrict the generalizability of the findings and make it challenging to draw firm conclusions regarding the effectiveness of incentive spirometer use. Many included studies had modest sample sizes and a moderate to high risk of bias, particularly related to randomization procedures, blinding, and control of confounding variables. Considerable heterogeneity in participant characteristics, COVID-19 severity, intervention parameters, outcome measures and comparator groups prevented quantitative synthesis through meta-analysis. In addition, variations in clinical practice, evolving COVID-19 management strategies, and incomplete or inconsistent reporting further limited interpretation of the findings. Finally, only a small number of studies assessed each outcome domain, which reduced the certainty and robustness of the conclusions. Future research should prioritize well-designed randomized controlled trials with standardized protocols, clear reporting of adherence, longer follow-up, and consistent outcome measures. Comparative studies evaluating different device types, combination therapies, and cost-effectiveness are also warranted to inform clinical practice.

## 5. Conclusions

Incentive spirometers are low-cost, portable devices that may support recovery in adults during the acute and Post-COVID-19 phases. self-management during COVID-19 recovery. Current evidence suggests that their use may improve pulmonary function, functional performance, and selected patient-reported outcomes; however, effects are inconsistent across outcomes and comparator interventions and are supported by low-to-moderate certainty evidence. Incentive spirometers may be most beneficial as an adjunct to respiratory physiotherapy within multimodal rehabilitation programs rather than as a standalone intervention.

While short-term use does not appear to influence severe clinical outcomes, such as mortality or intensive care admission, it may contribute to functional recovery and reduced length of hospital stay in selected patients. Given the limitations of the existing evidence, high-quality trials are needed to confirm effectiveness, establish optimal protocols, and guide clinical decision-making regarding the role of incentive spirometers in COVID-19 and Post-COVID-19 rehabilitation.

## Figures and Tables

**Table 1:** Search strategy used in each of the databases.

Sources	Search Strategy	Filters
PubMed	((“COVID-19” [Title/Abstract] OR “Post-COVID” [Title/Abstract] OR “Post-acute COVID-19 syndrome” [Title/Abstract] OR “long COVID” [Title/Abstract] OR “SARS-Cov-2” [Title/Abstract] OR (“SARS-Cov-2” [MeSH Terms] OR “COVID-19” [MeSH Terms])) AND (“incentive spiromet *” [Title/Abstract] OR “breathing exercise *” [Title/Abstract] OR “incentive spiromet * exercise*” [Title/Abstract] OR “Triflo” [Title/Abstract] OR (“respiratory exerciser” [Title/Abstract])) AND ((fft[Filter]) AND (english[Filter] OR greekmodern[Filter]))	Full text Language: English or Greek
Scopus	TITLE-ABS-KEY (COVID-19 OR COVID-19 pandemic OR COVID OR “post acute COVID-19 syndrome” OR “post COVID syndrome” OR sars AND Cov-2 OR “post COVID-19 syndrome”) AND TITLE-ABS-KEY (“incentive spirometer” OR “incentive spirometry” OR “breathing exercise” OR triflo OR “respiratory exerciser”)	Exclude: review, letter, editorial. Note, book chapter Language: English
CINAHL	AB (“incentive spirometer” OR “incentive spiroemetry” OR “breathing exercise” OR triflo OR “respiratory exerciser”) AND AB (“COVID-19” OR “COVID-19 pandemic” OR COVID-OR “post acute COVID-19 syndrome” OR “post COVID syndrome” OR SARS Cov-2 OR “post COVID-19 syndrome”)	Full text Language: English and Greek Exclude: electronic resources, reports and dissertations/Theses
PEDro	Abstract & Title (COVID-19 AND incentive spirometer) Abstract & Title (COVID-19 AND triflo) Abstract & Title (COVID-19 AND incentive spirometry)	Clinical Trial
Clinical Trials	(COVID-19 AND incentive spirometer) (COVID-19 AND incentive spirometry) (Post-COVID-19 Syndrome and Incentive spirometry) (Post-COVID-19 Syndrome and Incentive spirometer device) (Post COVID-19 Condition AND incentive spirometry)	With results
Google Scholar	COVID-19 (with exact phrase) AND “incentive spirometer” (with all of the words) Spirometry, post COVID-19 (with at least one of the words)	Anywhere in the article

* = indicates truncation of search terms.

**Table 2:** Characteristics of the included studies.

Author/ Year/ Country	Type of Study	Sample Size	Mean Age (SD)	COVID-19 Stage	Comparison Groups	Treatment Regimen	Outcomes
Kusumawardani et al., Indonesia, 2023 [58]	RCT	20	44.3 ± 6.8 48.1 ± 9.8	PCS	E: Incentive spirometer C: Diaphragmatic breathing exercise	E: 10 repetitions, 5× daily for 4 weeks C: 10 repetitions, 5× daily for 4 weeks	PEF
Gudivada et al., India, 2023 [59]	RCT	100	30.6 ± 6.2 31.9 ± 9.2	PCS	E: Incentive spirometer C: standard care (pharmacotherapy and behavioural therapy)	E: 10–15 repetitions every second awake hour C: not given	GAD-7 HARS PHQ-9 PFT
Oner Cengiz et al., Turkey, 2021 [53]	RCT	50	51.6 ± 14.2	Acute COVID with mild or moderate dyspnoea	E: incentive spirometer and standard care C: standard care	E: 5–10 repetitions per awake hour	SpO_2_ RR Dyspnoea-12 Questionnaire Beck anxiety inventory WHOQOL-Bref Number of hospitalisation days
Loganathan et al., India, 2022 [57]	RCT	24	37.3 ± 8.1 36.6 ± 10.2	PCS Mild to moderate symptoms	E: incentive spirometer and pharmacotherapy C: pharmacotherapy	E: 3 sets of 5 repetitions, every two awake hours, for 4 weeks	FVC FEV_1_ FEV_1_/FVC DLCO
Abo Elyazed et al., Egypt, 2024 [60]	RCT	60	39.8 ± 4.7 38.7 ± 4.2 40.4 ± 5.4	PCS with diaphragmatic dysfunction after mild to moderate infection	E1: incentive spirometer and standard care C: standard care	E1: 10–15 min, 2× daily for 8 weeks	mMRC dyspnoea scale 6MWT SVC FEV_1_/FVC MVV SpO_2_ Diaphragmatic thickness Diaphragmatic excursion
Bargahi et al., Iran, 2024 [54]	RCT	160	49.6 ± 12.2 47.7 ± 9.8	Acute COVID-19	E: Incentive spirometer and usual treatment C: usual treatment	E: 5 repetitions, 3 sets daily for 5 days to be continued for another 3 months	SpO_2_ VBG parameters MBS for dyspnoea LOS RR Mortality rate IR ICU admission rate
Harisuddin et al., Indonesia, 2023 [61]	Clinical trial (pre-post test control group design)	20	44.3 ± 6.8 48.1 ± 9.8	PCS	E: incentive spirometer C: Diaphragmatic breathing	E: 5 sets of 10 repetitions daily for 4 weeks C: 5 sets of 10 repetitions daily for 4 weeks	HRV RMSSD SDNN LF/HF ratio
Suharti et al., Indonesia, 2022 [55]	Clinical trial	45	47.2 ± 12.8	Acute COVID-19 Moderate to severe	E: incentive spirometer C: Diaphragmatic breathing	E: 10 repetitions, twice per day for at least 5 days C: 30 min per day for at least 5 days	4MGT 30STS
Tengker et al., Indonesia, 2022 [62]	Clinical trial (pre-post test control group design)	24	53.8 ± 6.1	PCS Mild to moderate symptoms	E: incentive spirometer C: Diaphragmatic breathing	E: 20 repetitions, twice daily, for 4 weeks C: 6 breath cycles, twice daily, for 4 weeks	PCF
Rantung et al., Indonesia, 2022 [63]	RCT	24	N/I (range: 40–65)	PCS Moderate symptoms	E: incentive spirometer C: Diaphragmatic breathing	E: 20 repetitions, twice daily, for 4 weeks C: 6 breath cycles, twice daily, for 4 weeks	6MWT
Zagoto et al., Indonesia, 2022 [64]	Clinical trial (pre-post test control group design)	11	N/I	PCS	E: incentive spirometer and diaphragmatic breathing C: Diaphragmatic breathing	E: 2 sets of 20 repetitions daily for 4 weeks and 3 sets, each of 5 min duration with 6 cycles per minute, twice per day for 4 weeks C: 3 sets, each of 5 min duration with 6 cycles per minute, twice daily for 4 weeks	6MST
Aydin et al., Turkey, 2022 [56]	Observational study	35	N/I (range: 39–75)	Acute COVID-19	E: incentive spirometer C: routine care	E: at least 4 times per day, 10 min per session	PEF

Abbreviations: E, experimental group; C, control group; control group; GAD-7, Generalized anxiety disorder-7; HARS, Hamilton Anxiety Rating scale; PHQ-9, Patient health questionnaire-9; PFT, pulmonary function test; PEF, Peak expiratory flow; FVC, forced vital capacity; FEV_1_, forced expiratory volume in 1 s; SpO_2_, oxygen saturation in the blood; RR, respiratory rate; WHOQOL-Bref, World Health Organization quality of life instrument short form; DLCO, Diffusion lung capacity for carbon monoxide; mMRC dyspnea scale, modified Medical Research Council dyspnea scale; 6MWT, 6 min walk test; SVC, slow vital capacity; MVV, maximal voluntary ventilation; VBG parameters, venous blood gas analysis; MBS for dyspnea, modified borg scale for dyspnea; LOS, length of hospital stay; IR, intubation rate; HRV, heart rate variability; RMSSD, root mean square of successive differences between normal heartbeats; SDNN, standard deviation of N-N intervals; 4MGT, 4 m gait time test; 30STS, 30 sit-to-stand test; PCF, peak cough flow; 6MST, 6 min step test; N/I, no information.

**Table 3:** Incentive Spirometer Parameters.

Author/ Year	Flow/Volume Oriented	Position	Hold of Inspiration	Overall Duration	Sets and Repetitions	Location
Kusumawardani et al., 2023 [58]	Flow oriented (TriFlo, Respiometer)	Sitting	3–5 s	4 weeks	5 sets of 10 repetitions, daily	Home
Gudivada et al., 2023 [59]	Flow oriented (Triflo, Teleflex Medical, Mexico)	NI	Not given	4 weeks	10–15 repetitions every second awake hour	Home
Oner Cengiz et al., 2021 [53]	Flow oriented (Triflo)	NI	NI	2–5 days	5–10 times every awake hour	Hospital
Loganathan et al., 2022 [57]	Flow oriented	Side lying	5 s	4 weeks	3 sets of 5 repetitions for every 2 awake hours, daily	Hospital
Abo Elyazed et al., 2024 [60]	Flow oriented (UNA01, Unicare, China)	NI	NI	8 weeks	10–15 min twice daily	Home
Bargahi et al., 2024 [54]	NI	Sitting or Semi-fowler	1 s	5 days	3 sets of 5 repetitions, daily	Hospital
Harisuddin et al., 2023 [61]	NI	NI	NI	4 weeks	5 sets of 10 repetitions per day	Home
Suharti et al., 2022 [55]	NI	NI	5 s	5 days	10 breaths, twice daily	Hospital
Tengker et al., 2022 [62]	Volume oriented	Sitting	5 s	4 weeks	20 repetitions twice daily	Home
Rantung et al., 2022 [63]	Volume oriented	Sitting	5 s	4 weeks	20 repetitions twice daily	Home
Zagoto et al., 2022 [64]	Volume oriented	NI	NI	4 weeks	20 repetitions twice daily	Home
Aydin et al., 2022 [56]	Flow oriented	NI	3 s	4 weeks	Session lasting no longer than 10 min, 4 times daily	Hospital

Abbreviations: NI, No Information.

**Table 4:** Summary of the Outcomes.

Author (Year)	Outcome	Baseline	Post-Intervention	Effect Size	*p*-Value *	Comparator	Baseline (Mean ± SD)	Post-Intervention	Effect Size	*p*-Value *	*p*-Value †
Kusumawardani et al. (2023) [58]	PEF (L/min)	394.00 ± 130.29	556.00 ± 152.25	d = 1.14	0.000	Diaphragmatic breathing exercise	450.50 ± 120.47	562.50 ± 130.06	d = 0.89	0.001	0.198
Aydin et al. (2022) [56]	PEF (mL/min)	Both groups were evaluated together, median discharge PEF was 225 (180–285) and the median follow-up PEF was 465 (312–512) with a difference of 175 (85–250).				Standard Care			-	-	0.304
Tengker et al. (2022) [62]	PCF	239.17 ± 48.70	362.50 ± 52.94	-	0.001	Diaphragmatic breathing exercise	219.17 ± 43.58	280.83 ± 55.83	-	0.001	<0.001
Rantung et al. (2022) [63]	6MWT	370 (350–410)	475.44 (455–525)	-	<0.001	Diaphragmatic breathing exercise	359.36 (320–405)	397.77 (370–475)	-	<0.001	<0.001
Zagoto et al. (2022) [64]	6MST	97.78 ± 15.39	134.11 ± 16.62	-	<0.001	Diaphragmatic Breathing exercise	92.73 ± 10,30	114.55 ± 19.94	-	0.002	0.009
Harisuddin et al., 2023 [61]	RMSSD (ms)	18.16 ± 12.85	18.56 ± 15.44	d = 0.09	0.781	Diaphragmatic breathing exercise	14.71 ± 5.58	16.58 ± 5.64	d = 0.67	0.060	0.379
	SDNN (ms)	28.79 ± 22.34	34.49 ± 27.89	d = 0.51	0.138		27.42 ± 8.09	28.30 ± 4.74	d = 0.12	0.722	0.269
	LF/HF ratio	2.80 ± 2.19	2.15 ± 1.96	d = 0.61	0.085		3.88 ± 1.75	3.77 ± 1.96	d = 0.10	0.750	0.272
Suharti et al. (2022) [55]	30STS	8.81 ± 0.56	13.52 ± 0.54	-	0.000	Diaphragmatic breathing exercise	12.14 ± 0.82	16.76 ± 1.21	-	0.000	0.0028
	4MGT	5.51 ± 0.47	3.45 ± 0.10	-	0.000		3.57 ± 0.46	2.65 ± 0.25	-	0.001	0.021
Loganathan et al. (2022) [57]	FVC (L%)	69.58 ± 4.27	80.25 ± 3.14	-	0.001	Standard Care	67.83 ± 5.65	71.75 ± 5.34	-	0.001	0.001
	FEV_1_ (L%)	71.50 ± 4.77	85.00 ± 5.06	-	0.001		70.67 ± 5.91	75.00 ± 6.01	-	0.001	0.001
	FEV_1_/FVC	102. 42 ± 1.31	105.42 ± 3.32	-	0.003		103.83 ± 2.66	104.08 ± 3.02	-	0.809	0.315
	DLCO (mL/min/mmHg%)	45.75 ± 7.02	60.25 ± 8.07	-	0.001		46.00 ± 4.37	50.00 ± 3.86	-	0.001	0.001
Gudivada et al. (2023) [59]	GAD-7	2.25 (0–3.5)	0 (0–0.5)	-	0.006	Standard Care	2 (0–3)	1 (0–2)	-	0.25	-
	HARS	2.5 (0.5–5.5)	0 (0–0.5)	-	0.013		2 (0–3)	2 (0–3)	-	0.10	-
	PHQ-9	2 (1–4)	0 (0–0.5)	-	0.006		1 (0–2)	0 (0–2)	-	0.12	-
Öner-Cengiz et al. (2021) [53]	BAI	25.32 ± 12.36	14.50 ± 7.41	-	<0.0001	Standard Care	26.05 ± 10.30	19.95 ± 13.02	-	0.003	0.285
	QoL	74.05 ± 7.42	77.82 ± 6.77	-	<0.0001		62.50 ± 15.97	65.95 ± 14.54	-	0.003	0.002
	LoS	-	3.04 ± 0.65	-	-		-	3.63 ± 0.90	-	-	0.014
	SpO_2_ (%)	92.77 ± 2.02	97.05 ± 1.46	-	<0.0001		92.23 ± 2.00	95.23 ± 1.11	-	<0.0001	<0.0001
	RR (per minute)	21.73 ± 2.49	18.09 ± 3.18	-	<0.0001		22.27 ± 3.17	19.36 ± 1.89	-	<0.0001	0.062
	Dyspnoea	21.32 ± 6.91	10.77 ± 4.52	-	<0.0001		22.82 ± 9.20	10.14 ± 5.61	-	<0.0001	0.07
Bargahi et al. (2024) [54]	SpO_2_	-	90.11 ± 5.9	-	-	Standard Care	-	90.10 ± 6.9	-	-	<0.001
	RR (per minute)	-	25.83 ± 6.58	-	-		-	24.55 ± 7.54	-	-	0.07
	Dyspnoea	-	1.46 ± 2.42	-	-		-	1.75 ± 2.89	-	-	<0.001
	LoS	-	6.5 ± 5.34	-	-		-	7.01 ± 4.84	-	-	0.001
	MR	-	3 (3.7)	-	0.47		-	5 (6.3)	-	-	0.05
	IR	-	3 (3.7)	-	0.47		-	5 (6.3)	-	-	0.06
	ICU rate	-	6 (7.5)	-	0.20		-	11 (13.8)	-	-	0.48
Abo Elyazed et al. (2024) [60]	SPO_2_	94.8 ± 0.8	97.9 ± 0.8	-	<0.001	Standard Care	94.7 ± 0.9	94.9 ± 0.8	-	0.29	<0.001
	Dyspnoea	1.9 ± 0.8	0.7 ± 0.7	-	<0.001		1.9 ± 0.7	1.7 ± 0.8	-	0.1	<0.001
	FEV_1_/FVC	80.4 ± 1.2	83.8 ± 2.9	-	<0.001		80.2 ± 1.9	80.8 ± 3.1	-	0.47	0.001
	SVC	75.1 ± 2.4	89.1 ± 3.1	-	<0.001		76.3 ± 2.1	83.1 ± 3.1	-	0.39	<0.001
	MVV	49.0 ± 4.5	51.1 ± 3.8	-	0.013		47.0 ± 4.9	47.7 ± 4.1	-	0.41	0.037
	Diaphragm Thickness	0.52 ± 0.11	1.04 ± 0.24	-	<0.001		0.56 ± 0.1	0.6 ± 0.27	-	0.62	<0.001
	Diaphragm Excursion	2.96 ± 0.18	4.53 ± 0.67	-	<0.001		2.93 ± 0.17	2.98 ± 0.61	-	0.71	<0.001
	6MWT	216.2 ± 46.8	396.9 ± 30.8	-	<0.001		202.6 ± 44.0	217.9 ± 30.4	-	0.22	<0.001

* = within-group (pre-post) analysis for the incentive spirometer group, † = between-group analysis (incentive spirometer vs. comparator). Presented as Mean ± standard deviation or Median (interquartile range). Abbreviations: FVC, forced vital capacity; FEV_1_, forced expiratory volume in 1 s; DLCO, Diffusion Lung Capacity for carbon monoxide; SpO_2_, blood oxygen saturation; RR, respiratory rate; PEF, peak expiratory flow; SVC, slow vital capacity; MVV, maximal voluntary ventilation; PCF, peak cough flow. GAD-7, generalized anxiety disorder-7; HARS, Hamilton anxiety rating scale; BAI, beck anxiety inventory; PHQ-9, patient health questionnaire for depression; QoL, quality of life; LoS, length of stay; 6MWT, six minute walk test; MR, one month Mortality rate; IR, intubation rate; ICU, intensive care unit admission rate; RMSSD, root mean square of successive differences between normal heartbeats; SDNN, standard deviation of N-N intervals; LF/HF ratio, Low frequency/high frequency ratio of heart rate variability; 4MGT, 4 m gait time test; 30STS, 30 s sit to stand test; 6MST, 6 min step test; d, Cohen’s D effect size.

## Data Availability

Not applicable.

## Supplementary Materials

The following supporting information can be downloaded at: https://www.mdpi.com/article/10.3390/jcm15041425/s1, Figure S1: Risk of bias by (a) domain and (b) study for randomized controlled trials; Figure S2: Risk of bias by (a) domain and (b) study for non-randomized controlled trials; Table S1: Certainty of Evidence (GRADE).

## Abbreviations

The following abbreviations are used in this manuscript:

COVID-19	Coronavirus 2019
SARS-CoV-2	Severe Acute Respiratory Syndrome Coronavirus 2
PHEIC	Public Health Emergency of International Concerns
ARDS	Acute Respiratory Distress Syndrome
PCS	Post-COVID-19 Syndrome
PRISMA	Preferred Reporting Items for Systematic Reviews and Meta-Analyses
SWiM	Synthesis without Meta-analysis
RCT	Randomized Controlled Trials
FVC	Forced Vital Capacity
FEV_1_	Forced Expiratory Volume in 1 s
DLCO	Diffusing Lung Capacity for Carbon Monoxide
SVC	Slow Vital Capacity
MVV	Maximal Voluntary Ventilation
PFT	Pulmonary Function Test
WHOOQOL-Bref	World Health Organization Quality of Life Instrument Short Form
mMRC	Modified Medical Research Council dyspnea scale
VBG parameters	Venous Blood Gas Analysis
MBS dyspnea	Modified Borg Scale for Dyspnea
HRV	Heart Rate Variability
RMSSD	Root Mean Square of Successive differences between normal Heartbeats
SDNN	Standard Deviation of N-N Intervals
LF/HF	Low Frequency/High frequency ratio of Heart rate variability
6MWT	6 Minutes Walk Test
6MST	6 Minutes Step Test
4MGT	4 Minutes Gait Time Test
30sSTS	30 s Sit To Stand test
ICU	Intensive Care Unit
pCO_2_	Mixed Venous Carbon Dioxide Pressure
SpO_2_	Blood Oxygen Saturation
RR	Respiratory Rate
PEF	Peak Expiratory Flow
PCF	Peak Cough Flow
GAD-7	Generalized Anxiety Disorder-7 Questionnaire
HARS	Hamilton Anxiety Rating Scale
BAI	Beck Anxiety Inventory
PHQ-9	Patient Health Questionnaire for Depression
QoL	Quality of Life
EQ-5D-5L	EuroQuality-5 Dimensions-3 Levels questionnaire for quality of life
DSI	Dyspnea Severity Index
LoS	Length of Hospital Stay
MR	One-month Mortality Rate
IR	Intubation Rate
IS	Incentive Spirometer
NI	No Information
