# Supplementary material for: Incentive Spirometer in COVID-19: A Systematic Review
Source: J Clin Med. 2026 Feb 11;15(4):1425. doi: 10.3390/jcm15041425 (PMC12941904; doi:10.3390/jcm15041425)
Supplement: Supplementary file 1 [file jcm-15-01425-s001.zip › jcm-4085048-supplementary.pdf]

Supplementary Material

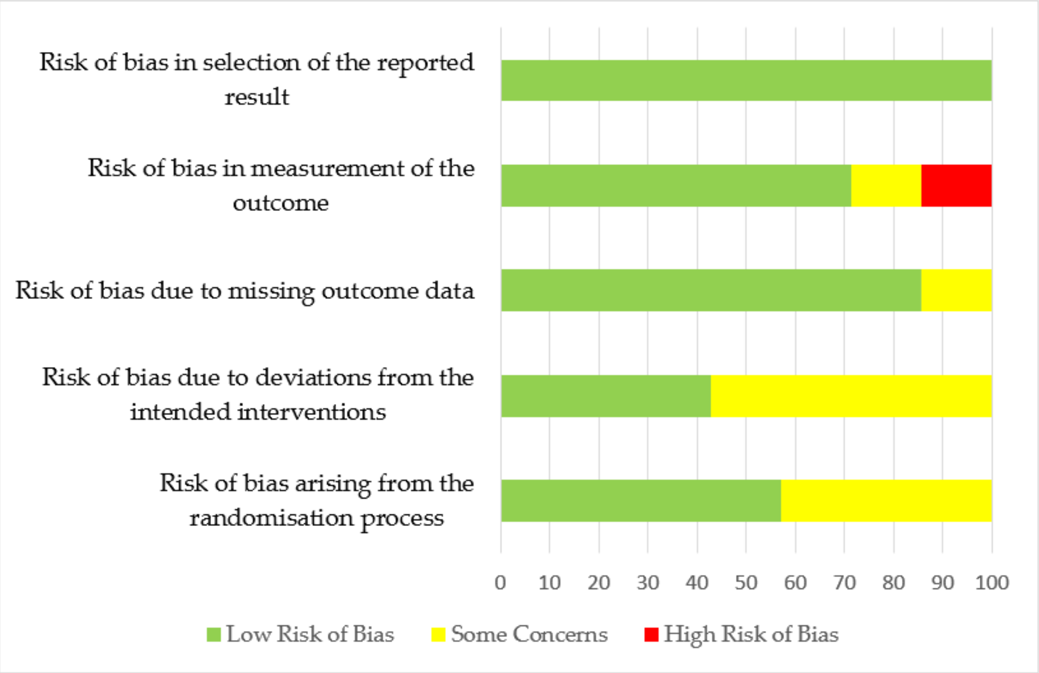

(a)

(b)

Authors (date)	Risk of bias arising from the randomisation process	Risk of bias due to deviations from the intended interventions	Risk of bias due to missing outcome data	Risk of bias in measurement of the outcome	Risk of bias in selection of the reported result
Abo Elyazed et al (2024)	+	+	+	+	+
Bargahi et al (2024)	+	+	+	+	+
Kusumawardani et al (2023)	−/+	−/+	+	+	+
Gudivada et al (2023)	+	+	+	−/+	+
Rantung et al (2022)	−/+	−/+	+	−	+
Loganathan et al (2022)	−/+	−/+	+	+	+
Oner Cengiz et al (2021)	+	−/+	−/+	+	+

Figure S1. Risk of bias by (a) domain and (b) study for randomized controlled trials. Domains were outlines as directed by the Cochrane Handbook for Systematic Reviews of Interventions. Green, yellow and red circles denote low, some concerns and high risk of bias, respectively.

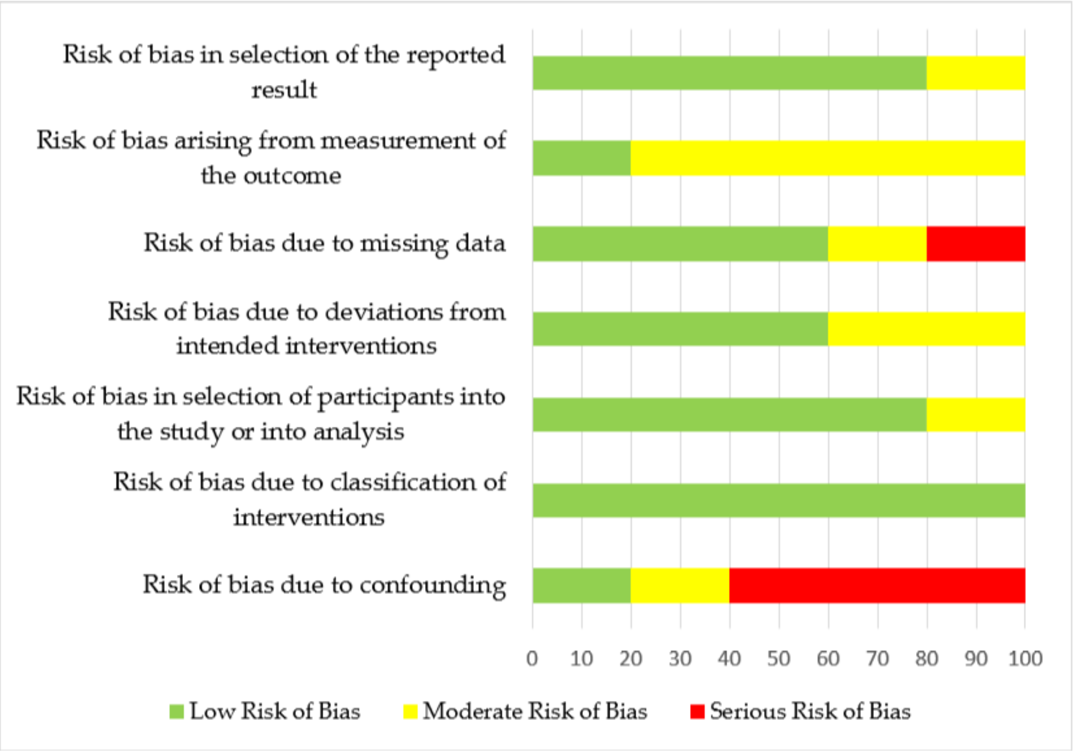

(a)

(b)

Author (date)	Risk of bias due to confounding	Risk of bias due to classification of interventions	Risk of bias in selection of participants into the study or into analysis	Risk of bias due to deviations from intended interventions	Risk of bias due to missing data	Risk of bias arising from measurement of the outcome	Risk of bias in selection of the reported result
Harisuddin et al (2023)	−	+	+	+	+	+	+
Suharti et al (2022)	+	+	−/+	+	+	−/+	−/+
Aydin et al (2022)	−/+	+	+	+	−/+	−/+	+
Tengker et al (2022)	−	+	+	−/+	+	−/+	+
Zagoto et al (2022)	−	+	+	−/+	−	−/+	+

Figure S2. Risk of bias by (a) domain and (b) study for non-randomized controlled trials. Domains were outlined as directed by Cochrane Handbook for Systematic Reviews of Interventions. Green, yellow and red circles denote low, moderate and high risk of bias, respectively.

Table S1: Certainty of Evidence (GRADE)

Outcome	Number of studies and design	Risk of bias	Inconsistency	Imprecision	Indirectness	Overall GRADE certainty
Pulmonary function	5 RCTs, 1 pre-post intervention, 1 observational	Moderate - High	Moderate heterogeneity	Moderate sample size	Direct	Low-Moderate
Dyspnea	3 RCTs	Moderate	Low heterogeneity	Moderate sample size	Direct	Moderate
Anxiety and Depression	2 RCTs	Moderate	Moderate heterogeneity	Moderate sample size	Direct	Moderate
Quality of life	1 RCT	Moderate	Not assessable	Small sample size	Direct	Very low
Functional tests	2 RCTs, 2 pre-post intervention	Moderate - High	Low heterogeneity	Moderate sample size	Direct	Moderate
Heart rate variability	1 pre-post intervention	Moderate	Not assessable	Small sample size	Direct	Very low
